# Neural Correlates of Changes in a Visual Search Task due to Cognitive Training in Seniors

**DOI:** 10.1155/2012/529057

**Published:** 2012-09-17

**Authors:** Nele Wild-Wall, Michael Falkenstein, Patrick D. Gajewski

**Affiliations:** ^1^Hochschule Rhein-Waal, University of Applied Science, Südstr. 8, 47475 Kamp-Lintfort, Germany; ^2^Leibniz Research Centre for Working Environment and Human Factors (IfADo), Ardeystr. 67, 44139 Dortmund, Germany

## Abstract

This study aimed to elucidate the underlying neural sources of near transfer after a multidomain cognitive training in older participants in a visual search task. Participants were randomly assigned to a social control, a no-contact control and a training group, receiving a 4-month paper-pencil and PC-based trainer guided cognitive intervention. All participants were tested in a before and after session with a conjunction visual search task. Performance and event-related potentials (ERPs) suggest that the cognitive training improved feature processing of the stimuli which was expressed in an increased rate of target detection compared to the control groups. This was paralleled by enhanced amplitudes of the frontal P2 in the ERP and by higher activation in lingual and parahippocampal brain areas which are discussed to support visual feature processing. Enhanced N1 and N2 potentials in the ERP for nontarget stimuli after cognitive training additionally suggest improved attention and subsequent processing of arrays which were not immediately recognized as targets. Possible test repetition effects were confined to processes of stimulus categorisation as suggested by the P3b potential. The results show neurocognitive plasticity in aging after a broad cognitive training and allow pinpointing the functional loci of effects induced by cognitive training.

## 1. Introduction

Spatial visual attention as for example, measured by a visual search task is highly relevant in everyday life, for instance when searching for an information sign in the environment, for a friend in a crowded place, or for specific items in a store. In laboratory studies visual search performance is mostly tested by presenting a target element in a display among different distractor elements. The target is more or less similar to the distractors, and is either present or not, which has to be indicated by the subjects. In such paradigms visual search performance was shown to decline with advanced age [1–3], especially, when the search task is difficult, for example, with high target distractor similarity (e.g., [4]).

Age-related decline in visual search performance as well as in other cognitive functions, for example, attention [5], occur with high interindividual variability [6]. Next to genetic predispositions the variability may be explained by a more or less supporting environment and lifestyle factors affecting the cognitive reserve, that is, the capacity to preserve intact fluid cognitive abilities in older age [7]. For instance education [8, 9], job demands [10, 11], free time activities [12], or physical fitness [13, 14] may prevent age-related cognitive decline.

Cognitive training is another possibility to maintain or even improve cognitive functioning in advanced age. Generally, cognitive trainings target specific and basic cognitive processes (cognitive process training: see [15]) with the idea that these processes assist a variety of higher order tasks to which transfer effects can be expected. Several studies showed the efficiency of specific cognitive process trainings in older participants for example, for working memory [16, 17], visual attention [18], dual task performance [19], visual discrimination [20], or speed of processing training [17, 21]. Also specific cognitive training in visual search performance was shown to be effective in older participants [17, 22]. However, compared to young, older participants show smaller training gains, are more susceptible to distraction [23], and seem to maintain a conservative search criteria, that is, increased response times, especially on target-absent trials [1, 24]. Additionally, a study by Fisk and colleagues [25] suggests that a training in semantic category search did transfer to other elements of the category in young participants but not in older ones. In general, the just cited studies mainly show training gains in the targeted cognitive skill. Evidence of near and far transfer for example, to other cognitive functions or even everyday functioning was less consistently reported. Nevertheless, other studies showed impressive far transfer effects to nontrained activities [26–30].

In the last decade broad cognitive trainings or “brain exercise” (based on PC, internet, or with paper and pencil) increased in popularity [31]. Many different tasks aim to target a variety of cognitive functions like for example, attention, short-term memory, dual-tasking, speed of information processing, or visual search. Effects of a multidomain training were less frequently studied although it is more likely that such training induces stronger transfer effects to non-trained or even daily life functions. Finally, multidomain training provides novelty, which most likely stimulates functional or even structural changes in the brain [32].

Evaluation studies are rare: in a training study including older adults, transfer was observed from a brain plasticity-based intervention (including speed of processing and working memory) to general memory performance [33]. A recent large population-based study by Owen and colleagues [34] found specific training effects to the trained tasks but no transfer effects even to closely related tasks. Therefore, it is unclear whether visual search performance can be efficiently improved by a broad cognitive training in older participants and if it shows at least near transfer to a search task which was not specifically practiced.

In the present study we aimed at evaluating if visual search performance can be efficiently trained in older participants by a multilayered cognitive training intervention. In addition we aimed at identifying the basic cognitive processes which underlay a possible training gain in a near transfer task. To this aim, we setup a multidimensional cognitive process training targeting a wide range of basic cognitive processes like selective attention, working memory, short-term memory, reasoning, speed of processing, mental rotation, and vigilance. The training was administered to senior participants during a four-month interval. Training effects should be distinguished from retest effects and from effects of social interaction by including two control groups: a no-contact control group and a social control group which received a relaxation training during the same time as the cognitive training group. Initially, all participants were tested on a visual search task and other cognitive tasks (which will be presented elsewhere) not being part of the training intervention. These tasks were conducted to allow the investigation of transfer effects. The visual search and the other cognitive tasks were again administered after the four month period.

Although it is assumed that training interventions boost functional or even plastic changes to the brain, neuronal correlates of the training induced changes in intervention studies were only examined in the last decade [35, 36] and were shown to occur also in advanced age (see [37] for a review). For instance, Mozolic and colleagues [38] aimed at improving attention and reducing distractibility in older adults with a cognitive training intervention and showed that the resting cerebral blood flow in prefrontal regions was increased. This increase was related to better task performance after training. Knowledge about the intervention related neuronal and functional changes is additionally useful in order to understand the efficiency of the training and transfer effects to other tasks [15]. Therefore, in the present study we used event-related brain potentials (ERPs) derived from the electroencephalogram (EEG) in order to study more closely the neuronal processes which are affected by the training intervention.

For the present study we hypothesized that the cognitive training group which was trained on a variety of cognitive processes, would improve their performance in a visual search transfer task. Based on the cited literature, we expect training effects for reaction times and/or for error rates (or missed targets), thus, reflecting a gain in efficiency for visual search performance in the cognitive training group, suggesting transfer to a not explicitly trained task.

Different ERPs were used in order to pinpoint the functional processes which would be improved by the cognitive process training and which may be affected by retesting. The principal ERP components elicited after task-relevant visual stimuli are among others the N1, the anterior N2, the P2, and the P3b. The N1 potential, measured over occipital scalp regions, is thought to reflect sustained covert attention during visual processing [39]. The N2 is also frequently measured in visual search paradigms [40]. In the present study we more specifically analyzed the anterior N2 as an index of cognitive control [40, 41]. The P2 is a positive potential that peaks around 200 ms and is maximal over the vertex [42, 43]. In the visual domain the frontal P2 is sensitive to the detection of stimulus features like orientation or colour [44]. The P2 has been used in visual search paradigms as a correlate of visual feature discrimination [45]. Thus, in the present study we take the P2 potential as an index of attentive and feature based stimulus processing which should be more pronounced with increasing stimulus relevance [46]. The P2 is sensitive to performance improvements after training of simple stimulus discrimination in the visual domain [35, 47]. According to these previous results of others we assumed that the expected training gains may be due to improved feature selective attention which should hence be reflected in an increased P2 amplitude and/or decreased P2 latency.

We also examined the P3b potential, which can be measured as a broad positive peak over centro-parietal scalp regions. A recent influential theory by Polich [48] relates this potential to memory-based stimulus categorisation. According to this theory, the parietal P3b can be seen as a correlate of a categorisation process of task relevant stimuli which follows stimulus evaluation by earlier attention-driven working memory processes. For the P3b we speculated that the cognitive training may also improve processes of stimulus categorization which should hence be reflected by an increased P3b amplitude to targets after the training.

We also aimed at distinguishing the training gains from retest effects on the level of brain functions. Therefore, we randomly assigned the participants to a cognitive training group, a social control group and a no-contact control group. We speculate that retest effects might rather affect later stages of stimulus processing as a consequence of being familiar with the task and response option. Such effect would apply to all groups and could be manifest in improved stimulus categorisation, as reflected by the P3b, and reduced response times. In contrast, the cognitive training could additionally affect earlier processing stages as a consequence of an improved search strategy and improved attentional processes. This should be reflected in an even higher performance gain of the cognitive training group compared to the control groups and possibly affecting the P2 potential as was found in prior training studies [22, 23].

## 2. Method

### 2.1. Participants

One hundred and fourteen participants, aged between 65 and 88 years, were recruited to participate in the experimental study which was conducted in Dortmund, Germany. The participants were randomly assigned to the training groups. Thirty nine of them represented a cognitive training group (remaining *N* = 32; 12 men, mean age: 70.5 years; range 65 to 82; seven drop-outs because of technical problems, illness, and tenancy changeover). The other participants formed a no-contact control group (*N* = 39; 16 men, mean age: 69.7 years; range: 65 to 88; no drop-outs) and a social control group (remaining *N* = 34; 13 men, mean age: 70.9 years; range: 65 to 87; two dropouts because of illness). The cognitive training group was exposed to a multilayered cognitive training over a periode of 4 month. At the same time, the social control group received a relaxation training. All groups were comparable in age, education, and in their cognitive status assessed by Mini Mental State Examination (MMSE german version: [49]), verbal IQ (MWT-B: [50]), forward and backward digit repetition, and Trail-Making test A and B (see Table 1 for details).

### 2.2. Cognitive and Relaxation Training

The participants in the two training groups trained twice a week for 90 minutes across 4 months. Both trainings were conducted in small groups with not more than 12 participants by payed professional trainers. Two extra sessions were offered at the end of the program for those participants who missed the regular sessions. The participants were not encouraged to train outside the training sessions. The relaxation training of the social control group included toning, muscle stretching and relaxation, correct breathing, autogenic training, and education about a healthy lifestyle.

In the cognitive training group a mental activation training (MAT: [51]) was used in the first four weeks. The MAT is a paper and pencil package with short exercises to increase working memory capacity, visual attention, and speed of processing. Additionally, in this first eight sessions participants without any PC-experience were made step by step familiar with the computer mouse and keyboard. In the following weeks, the participants trained using selected commercial and noncommercial internet-based software (http://www.freshminder.de/; http://www.mentaga.de/; http://www.ahano.de/; http://www.mental-aktiv.de/). Each session consisted of different exercises aimed at training crucial cognitive functions: attention, working memory, short term memory, speed of processing, reasoning, and verbal meaning. The exercises were relatively complex and involved mainly attentional and mnemonic functions. No explicit visual search exercise was included in this program. In particular the training package FreshMinder includes games that require fast responding to specific stimuli like colored balloons, fast selecting digits in ascending order, memorizing and delayed recall of faces, repeating of sound sequences, matching of letters, shopping lists, counting of bricks in 3-D figures, and memorizing and recall of schematic paths. Ahano peds consists of units with different levels of difficulty. The free available program includes an eye-hand coordination task, money counting task, detection of word repetitions in a text, block taping task, and memory for abstract figures. Mentaga consists of exercises enhancing vigilance, perceptual speed, spatial cognition, and so forth. Finally, mental-aktiv offers a number of memory tasks using digits, letters, colors and figures, and exercises to train speed of processing.

The participants were not encouraged to exercise outside the training sessions but to continue the training at home after the study was finished.

### 2.3. Procedure of Cognitive Testing

Before and after the training interventions, all groups received a before and after test session, respectively. Before the first session participants filled at home a number of sociodemographic questionnaires. A neuropsychological testing using paper and pencil tests and a PC-based testing during the EEG-recording were completed at two different days. During the EEG-session several cognitive tests were performed in order to determine a comprehensive cognitive status. In the current study only the visual search task is reported, data from the other tasks will be reported elsewhere.

For cognitive testing, participants were seated in a dimly lit sound attenuated room, 60 cm in front of a CRT-monitor. The visual search task was a conjunction search task with two possible targets. Participants had to search a target in a three by three array consisting of green and red arrows presented on a dark grey background. A green arrow pointing upwards and a red arrow pointing rightwards were defined as target stimuli. The distractor arrows were red and green as well and pointing in the remaining directions. The task included a total of 104 trials. A target occurred in 50% of the trials. Participants were instructed to press a response button as fast as possible with the right index finger only if a target was detected in the array. A special response pad including only the response button was used and connected with a PC over a game port in order to guarantee the recording or the response in real time.

A trial started with a white fixation point which was presented in the middle of the screen for 1 second. Following, the 3 × 3 search array was presented for three seconds which was the maximal response window (in case of a target). The next trial followed immediately. A single arrow was about 9 mm high and 5 mm wide (0.8 by 0.44 degrees visual angle). The whole search array spread an area of about 30 by 30 mm, resulting in a visual angle of 2.6 by 2.6 degrees.

### 2.4. Data Recording and Analysis

#### 2.4.1. Electrophysiological Recording

 The Electroencephalogram (EEG) was recorded from 32 active electrodes positioned according to the extended 10–20 system [52] and mounted on an elastic cap (the electrodes mounted in the cap included the following positions: C3, C4, CP3, CP4, CPz, Cz, F3, F4, F7, F8, FC3, FC4, FCz, Fp1, Fp2, Fpz, Fz, O1, O2, Oz, P3, P4, P7, P8, PO3, PO4, POz, Pz, T7, and T8.). Electrodes M1 and M2 were placed at the left and right mastoids. The horizontal and vertical EOG was measured by electrodes placed at the outer canthi (LO1, LO2) and above and below both eyes (SO1, SO2, IO1, IO2). Electrode impedance was kept below 10 kOhm. The amplifier bandpass was 0.01–140 Hz. EEG and EOG were sampled continuously with a rate of 2048 Hz. Data were saved on a hard disc alongside with triggers marking significant events.

Offline, the EEG was downscaled to a sampling rate of 500 Hz and cut in stimulus locked epochs by using the software Vision Analyzer (Brain Products, Munich, beaming). The epochs were 1200 ms long ranging from 100 ms before and 1000 ms after stimulus onset. All epochs with EEG amplitudes of more than ±120 *μ*V or with drifts of more than 150 *μ*V within 300 ms were discarded. A regression based method was used for eye movement correction [53] by using the horizontal (LO1 versus LO2) and vertical (SO1 versus IO1) EOGs. For all participants and conditions at mean 48 epochs (Min = 17; Max = 53; SD = 7.3) of the epochs remained for averaging after artefact rejection and correction. The epochs were averaged according to the stimulus conditions (target trials versus nontarget trials) and rereferenced to linked mastoids (excluding the EOG electrodes). For stimulus locked averages only correct epochs were used, excluding trials with false alarms or misses. A digital low-pass filter was set at 17 Hz.

#### 2.4.2. Statistical Analysis

 Statistical analyses were performed by means of repeated measures ANOVAs with Greenhouse-Geisser corrected degrees of freedom. In case of significant main effects (if the factor included more than two levels) or interactions additional ANOVAs were applied for post hoc testing of contrasts and simple effects.

For response times (RTs; correct commission trials) the ANOVA included the within factor *time* (session one, session two) and the between factor *group* (cognitive training group, social control group, no-contact control group). Separate ANOVAs were carried out for false alarms and for misses, because they are different types of errors either demanding a response or not. Both analyses included the factors *time* and *group*.

The peak amplitude and latency of the N1 potential was measured at the two occipital electrodes O1 and O2 were the potential showed its maximum. The N2 was quantified as the mean amplitude in the time interval between 240 to 300 ms at the electrodes FCz, Cz and CPz were it showed the maximum amplitude. A reliable measurement of the peak was not possible due to the overlapping P2, and P3b potentials. The P2 potential was quantified in amplitude and latency as the local maximum at the electrodes FCz, Cz and CPz in the search interval between 200 and 400 ms where it showed the highest peaks. The peak amplitude and latency of the P3b potential was measured as the local maximum at the electrodes Cz, CPz and Pz in the search interval between 400 and 700 ms where it showed the highest amplitudes.

Six separate ANOVAs were carried out for the peak amplitudes and latencies of the N1, P2 and the P3b, respectively, including the between subject factor *group* and the within subject factors *session *(session one, session two), *stimulus type* (target, nontarget) and *electrodes *(O1 and O2 for the N1; FCz, Cz, and CPz for the P2 potential; Cz, CPz, and Pz for the P3b potential, resp.). An additional ANOVA was carried out for the N2 mean amplitudes including the between subject factor *group* and the within subject factors *session*, *stimulus type,* and *electrodes *(FCz, Cz, and CPz).

We use sLORETA [54] in order to closer examine the underlying neuronal changes of the expected training effect of stimulus feature processing as reflected by the P2. We examined only the target condition because the training gains may especially help to improve target detection. The program sLORETA estimates the sources of activation on the basis of standardised current density at each of 6239 voxels in the grey matter of the MNI-reference brain with a spatial resolution of 5 mm. The calculation is based upon a linear weighted sum of the scalp electric potentials with the assumption that neighbouring voxels have a maximal similar electrical activity. The voxel-based sLORETA images were first computed for each individual averaged ERP in the target condition in the interval from 170 to 190 ms surrounding the P2 peak. Then, the differences of the sLORETA images between test sessions were statistically compared between groups using the sLORETA voxelwise randomisation test (5000 permutations) which is based on statistical nonparametric mapping (SnPM) and implemented in sLORETA. Two independent group tests were carried out for comparison of the three groups (cognitive training group versus no-contact control groups, and versus social control group). The tests were performed for an average of all time frames in the interval with the null hypothesis that (T1_groupA_  − T2_groupA_)  = (T1_groupB_ − T2_groupB_). The tests were corrected for multiple comparisons [55].

## 3. Results

### 3.1. Performance Data

#### 3.1.1. Response Times

The mean RT to target stimuli was 1354 ms (see Table 2 for details). The factor *time* nor any other factor or interaction reached significance (all *P*s > .15).

#### 3.1.2. False Alarms

 False alarms to nontargets (Table 2) were committed in about 3.2% of the trials. There were no significant effects of *time* or *group* nor was there an interaction.

#### 3.1.3. Misses

 Targets were missed in about 23.5% of trials. There was a significant interaction of *time *×  *group* (*F*(2,102) = 3.1, *P* = .05): while the cognitive training group significantly reduced the rate of misses from the bafore (25.7%) to the after test (19.9%; *F*(1,31) = 2.2, *P* = .012), no significant changes were found for the social control group (28.4% versus 26.1%; *P* = .18) or the no-contact control group (23.9% versus 24.5%; *P* = .73).

### 3.2. Electrophysiological Data

#### 3.2.1. N1

**Figure 1:**
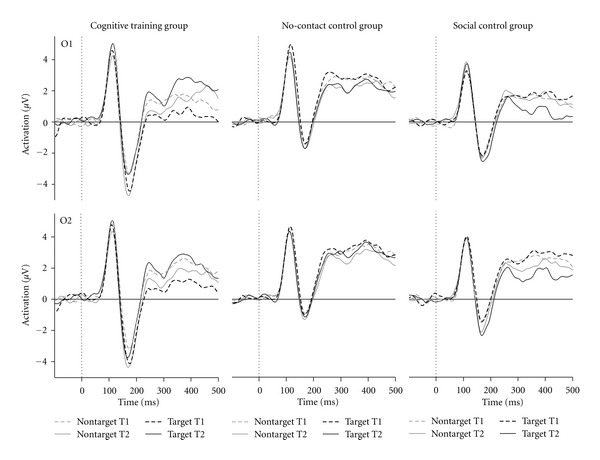
Stimulus-locked event-related potentials at the occipital electrodes O1 and O2 separately for target and non-target trials, for the first (T1) and the second test session (T2) as well as for the cognitive training group, the no-contact control group and the social control group. Note that the N1 is the pronounced negative peak in the interval between 150 to 200 ms.

Analysis of the N1 amplitude at electrodes O1 and O2 (see Figure 1) revealed a significant three-way interaction of *session* x  *stimulus type* x *group* (*F*(2,102) = 3.22, *P* = .044). This was due to an increased N1 amplitude from the before to the after session only for the cognitive training group and only for the non-target stimuli (−4.5 *μ*V versus −5.6 *μ*V: *F*(1,31) = 10.1, *P* = .003). In addition, in the after session, the non-target N1 amplitude was significantly higher for the cognitive training group (−5.6 *μ*V) when compared to the social controls (−2.2 *μ*V; *P* < .001; Bonferroni corrected) or to the no-contract control group (−2.9 *μ*V; *P* = .002; Bonferroni corrected). No other effects for the N1 amplitude reached significance nor any effect for the N1 latency.

#### 3.2.2. N2

**Figure 2:**
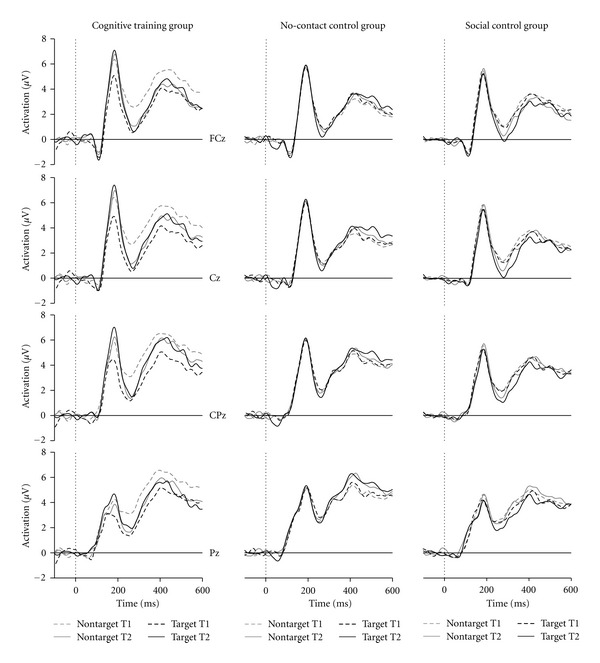
Stimulus-locked event-related potentials at selected midline electrodes separately for target and non-target trials, for the first (T1) and the second test session (T2) as well as for the cognitive training group, the no-contact control group and the social control group. Note that the P2 is the first pronounced positive peak with a maximum at Cz, and the P3b is the second broad positive peak with maximal amplitudes at CPz and Pz.

 The N2 (see Figure 2) showed a maximum at the electrodes FCz (1.2 *μ*V) and Cz (1.4 *μ*V) and was less negative at CPz (2,1 *μ*V; main effect of f *electrodes*: *F*(2,204) = 30.7, *P* < .001). The tree-way-interaction of the factors *session* x *stimulus type* x *group* reached only a trend (*F*(2,102) = 3.01; *P* < .06).

#### 3.2.3. P2

 Descriptively, the P2 showed a broad central topography with highest amplitudes at Cz (about 7.4 *μ*V; see Figure 2). There was a significant three-way interaction of *time* x *stimulus type* x *group* (*F*(2,102) = 3.5, *P* = .036): The P2 after target stimuli increased from the before to the after test only for the cognitive training group (7.1 versus 8.8 *μ*V;* F*(1,31) = 6.5, *P* = .016) but not for the social control group (6.6 versus 6.6 *μ*V) nor for the no-contact control group (7.3 versus 7.4 *μ*V). No significant differences were observed for the P2 amplitude after non-targets from the before to the after session nor between groups.

The P2 latency (Figure 2) did not change from the before to the after session. Generally, the potential peaked earlier at Cz (187 ms) and CPz (185 ms) compared to FCz (189 ms; both *F*s(1,102) > 6.5, both *P*s < .013; resulting in a main effect of electrode: *F*(2,204) = 5.6, *P* = .015).

**Figure 3:**
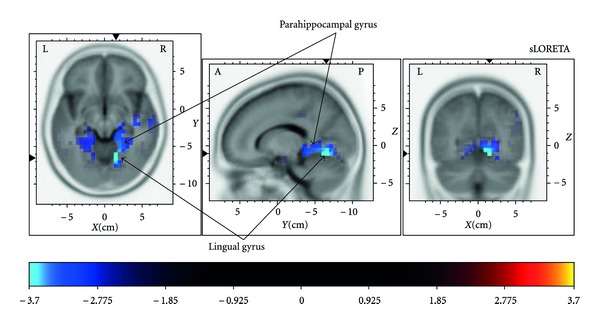
Graphical representation of the sLORETA results comparing the differences of the target-P2 between test sessions exemplarily for the independent group test including the cognitive training group and the no-contact control group. Similar results were obtained when including the social control group. The blue colour indicates local maxima of lower activation in the first compared to the second test session for the cognitive training group in the right lingual and parahippocampal gyri, which may explain the amplitude difference of the P2 between sessions in the tested interval surrounding the P2 peak.

In order to examine the underlying neuronal changes of the training gain as reflected in the P2 amplitude, the source for the P2 amplitude difference was examined in the time interval from 170 to 190 ms surrounding the potentials peak (see method section for details). The analysis points mainly to two connected brain regions in Brodmann area 19, the lingual and parahippocampal gyri (see Figure 3) which showed a significantly higher activation after the cognitive training compared to before in the training group (Talairach coordinates: (*x*) 14 to 20, (*y*) −51 to −70, (*z*) −6 to −12; *t*s < −3.2, *P*s < .05). Both control groups showed no significant change in activation between test sessions in any brain region.

#### 3.2.4. P3b

 The P3b had a centroparietal maximum (*electrode*: *F*(2,204) = 41.9, *P* < .001) with highest amplitudes at Pz (7.2 *μ*V) and CPz (7.0 *μ*V) and lower amplitudes at Cz (6.0 *μ*V; both *F*s(1,102) > 44.8, both *P*s <.001) which confirmed that it is the P3b. The interaction of *time* x *electrode* x *stimulus type* was significant (*F*(2,204) = 5.9, *P* = .005): At the presession the P3b for targets was highest at Pz (7.0 *μ*V), lower at CPz (6.7 *μ*V) and again lower at Cz (5.7 *μ*V; all *F*s(1,102) > 4.4, all *P*s <.038), however, for non-targets amplitudes did not differ at CPz (7.1 *μ*V) and Pz (7.3 *μ*V) and were lower at Cz (6.1 *μ*V; both *F*s(1, 102) > 38.8, both *P*s <.001). At the after session, the P3b for non-targets showed a decrement from Pz (7.3 *μ*V) over CPz (6.8 *μ*V) to Cz (5.7 *μ*V; all *F*s(1,102) > 7.4, all *P*s<.01) whereas for targets it was similar at Pz (7.4 *μ*V) and CPz (7.3 *μ*V) and was significantly lower for Cz (6.4 *μ*V; all *F*s(1,102) > 16.0, all *P*s <.001). Thus, whereas the P3b showed a more posterior amplitude distribution for non-targets after the training, the distribution was more anterior for target stimuli. No other main effect nor an interaction reached significance or a trend.

Whereas the P3b peak latency did not differ between the electrodes in the presession, it peaked earlier at Pz (533 ms) compared to CPz (543 ms; *F*(1,102) = 7.4, *P* = .008) in the after session (interaction of *time* ×  *electrode*: *F*(2,204) = 3.7, *P* = .031). No other effect reached significance.

## 4. Discussion

In the present study we found evidence for the efficiency of a broad cognitive training for improving the performance of older participants in a near transfer task of visual conjunction search. More specifically, our experimental group showed a reduced rate of missed targets in the after training session compared to the session before the training. On the other hand, the response times and the false alarm rate were not affected by any experimental factor. Therefore, the improved performance in the cognitive training group, that is, the lower rate of missed targets, cannot be explained by a more liberal response criterion (the calculation of a sensitivity parameter is problematic, because several participants in the three groups do not have any false alarms) or a speed-accuracy trade-off. Although the training intervention also included aspects of visual search, the training tasks and the tested task were completely different concerning the stimuli and processing demands. Hence the performance improvement in the training group suggests near transfer. Both control groups did not show any significant improvements of their performance in the visual search task. Therefore, the observed effect in the cognitive group cannot be explained by test repetition (in comparison with the no-contact control group) or by social interaction (in comparison with the social control group receiving relaxation training). Our results are in line with previous training studies which also found evidence for improvements of specific cognitive functions after cognitive training in older participants (e.g., for working memory: [16], e.g., for dual task performance: [19]).

Performance improvements of older participants were also found for training of visual conjunction search in other training studies [23, 24]. These studies found evidence that the older participants learn almost as good as the young ones to efficiently use feature information to selectively attend to those objects in the search array that share common features with the target. Our findings go further and show the neuronal correlates of the functional processes which likely were improved by the training: In the cognitive training group the occipital N1 was enhanced after versus before the training for nontarget stimuli. This suggests that the participants developed mechanisms for enhanced attention of arrays which were not immediately recognized as targets, that is, the nontargets. The frontal N2 to nontargets was also increased in amplitude for the cognitive training group after training. However, as this effect failed to reach significance, it can only be speculated that also the subsequent processing or even inhibition of the nontarget stimuli improved after cognitive training. Based on the enhanced attention in nontarget trials in the cognitive training group as was reflected in the N1 amplitude, one may expect also a decrease in the false alarm rate. However, the lack of effect on the false alarms may not be surprising due to the generally low rate of false alarms in all groups. A weak hint may give the nonsignificantly but numerically lower rates of false alarms in the after compared to the before session in the cognitive training group whereas the two control groups showed a numeric increase.

The increased amplitude of the P2 in target trials may suggest that feature based stimulus processing was improved in our older participants after the cognitive training. Consequently, the improved discrimination of stimulus features in target-present trials should decrease the likelihood of missed targets and increase the likelihood of target detection. This effect on performance data was evident in our cognitive training group after the training compared to the pretraining session and when compared to the control groups.

The sLORETA analysis of the P2 amplitude differences between test sessions elucidates the neuronal basis of the training gain. Specifically, activation in the lingual and parahippocampal gyri was increased only in the cognitive training group and not in the two control groups. Most importantly, the increased P2 amplitude together with the significant changes in brain activation show that the cognitive training caused a change in brain processes on a functional level in a near transfer task of visual search. Both regions are anatomically and functionally connected [56] and are discussed as being sensitive for global visual feature processing [57], as well as the global processing of spatial layout [58] and surface properties like color and texture of scenes and objects in visual arrays [56]. For our training group this may mean that the cognitive process training improved the textual and spatial processing of visual arrays in general. Possibly, the use various kinds of visual material like pictures, objects, and text pages which were used in various tasks in the training sessions did improve one basic cognitive process of global processing of visual arrays. The present results also suggest the P2 potential of the ERP as a possible marker for the improvement of this cognitive process.

In the present study we were able to distinguish the functional processes which were sensitive to the training intervention from retest effects. In fact, the effect of test sessions on the topography of the P3b applies to all groups. We assume that the P3b may reflect memory-based stimulus processing. Thus, whereas attentional processing of target-absent trials (N1 results) and feature-based stimulus processing of target-present trials (P2 results) were only modulated by the cognitive training intervention, the improvement of stimulus categorisation, which is based on memory representations (P3b), was sensitive to retesting. In a previous study, Roche and O'Mara [59] found that the learning of a stimulus-response (S-R) association improved the performance in a simple visual search task which included the same S-R association. In addition, the P3b amplitude increased and latency decreased in their trained participants but not in a control group as a reflection of the learned S-R association. Thus, it is possible, that the modulation of the P3b topography for target stimuli in the present study may also reflect an improvement in stimulus-response learning from the before to the after test for all groups. The mere test repetition may be sufficient to learn this simple S-R association in the present study which consisted of only one button press in case of target-present trials. In contrast to Roche and O'Mara [59], the present data did not reveal a reduced latency of the P3b with learning. The P3b latency was rather slightly increased from the before to the after session, which parallels the slight RT increase. This adds to the evidence that the P3b contains a component related to response selection or execution [60, 61]. Nevertheless, it is possible that in the present study the test repetition improved the efficiency of S-R association. Latency improvements in RT or P3b due to S-R learning, as was the case in the study by Roche and O'Mara [59] are possibly counteracted by several reasons: (i) the generally long response times well above 1000 ms which are possibly due to a generally conservative search criterion of our older participants, (ii) a thorough search strategy in the cognitive training group, leading to numerically increased response times and, (iii) the long interval of 4 month between the before and after session which may have weakened the S-R association.

Interestingly, the P3b did not differentiate between target and non-target trials in the present study. Normally, the P3b is increased for targets compared to non-targets in various paradigms in the auditory and visual domain (e.g., [48]). This can be interpreted as the allocation of processing resources to relevant stimuli. The lack of difference between conditions may suggest that the target-absent trials received as much processing resources as the relevant target-present trials. Two interrelated reasons are suggested to explain the present data: (i) the discrimination of target-present and target-absent trials is very difficult leading to an increased variability of the discrimination process, to a postponement of the decision and consequently smearing out the P3b and (ii) due to a conservative search criterion the decision to categorize the trial as an target-absent trial or vice-versa is postponed by the subject leading to delay in the processing which falls outside the time range of the P3b. The two suggested reasons would apply to all groups, and thus, stimulus categorisation was not specifically improved by the cognitive training intervention. The interpretation of the P3b is limited because by visual inspection (see Figure 2) it seems that the amplitude is reduced from the before to the after session for the cognitive training group in the non-target condition. This, however, is not reflected in any significant effect in the ANOVA. In addition, it may result from an overlap of the preceding N2 potential.

Our study bears several shortcomings which may give directions for further studies. First of all, the presented visual search task was the only implemented transfer task which included aspects of visual search. Therefore, we are not able to validate the improvement of visual search performance by the cognitive training with results in another transfer task. In addition, the cognitive training was multidimensional and aimed mainly at enhancing basic and executive functions tested by a number of our tasks in order to improve daily life activities. As the training was domain unspecific, it is not possible to show divergent results in two or more tasks in the effects of the training procedure. Further studies which aim to evaluate broad cognitive trainings should bear in mind (i) to use more than one transfer task which assess the same cognitive function in order to show convergent effects of the training and/or, (ii) to use transfer tasks assessing cognitive functions which were not intended to be improved by the training in order to show divergent effects. Another shortcoming of the present study is that participants showed very long response times and very low rates of false alarms due to high complexity of the task and moderate time pressure of three seconds. Therefore, it was not possible to calculate any sensitivity parameter which would have further underlined the interpretation of the present results to reflect the improvement of visual feature processing. Further studies could include the presentation of more ambiguous stimuli within a visual search paradigm or to give time pressure in order to decrease response times and therewith the rate of false alarms. An additional shortcoming of the present study is the fact that only the cognitive training group received basic PC-practice which may have made them more experienced with computer technology than the other groups. However, although computerized testing took place in the before and after test sessions, the interaction of the participants with the PC was reduced to a minimum and the manual responses were collected with special response buttons and not with a computer keyboard or a mouse. In addition, the search array of the transfer task comprised only 2.6 degrees of the visual angle which is not comparable to the input of an entire computer screen. Therefore, we do not think that the basic PC-training of the cognitive training group may explain the transfer effects in the visual search task. Further training studies should try to exclude any confounding effect of the training procedure on the evaluation of the training effects.

## 5. Conclusion

To summarise, in the present study we found evidence for the efficiency of a broad cognitive training for improving the performance of older participants in a near transfer task of visual conjunction search. The electrophysiological data helped to elucidate the functional processes which were sensitive to the training intervention and, on the other hand, to retest effects due to task repetition. Additionally, the mediating neuronal basis of the training gain was identified, thus, underlining the efficiency of the training to induce functional changes in the brain. More specifically, the cognitive training especially improved the global feature processing of visual arrays which may explain the improvement in target detection within a given time window in the near transfer task of visual conjunction search. These results cannot be explained by test repetition or by the mere social interaction of the training intervention, suggesting that a multilayered formal cognitive training is sufficient to facilitate neuronal plasticity in older age.

## Figures and Tables

**Table 1:** Demographic characteristics and cognitive status of the participant groups (Mini Mental State Examination, verbal IQ, forward and backward digit span, and Trail-Making Test A and B). Standard deviations are given in parentheses behind the mean values. There were no significant group differences as is indicated by the statistical analyses (last column).

Group	Cognitive training	No-contact control	Social control	
Mean age	70.5 years (4.3)	69.7 years (4.5)	70.9 (4.4)	*F*(2, 102) = 1, *P *= .36
MMSE score	28.8 (1.9)	28.1 (1.9)	28.5 (1.6)	*F*(2, 102) = 1.4, *P *= .24
Verbal IQ (MWT-B)	116 (11.5)	118 (13.9)	117 (11.7)	*F* < 1
Forward digit repetition	3.6 (0.6)	3.7 (0.7)	3.8 (0.8)	*F* < 1
Backward digit repetition	2.9 (0.6)	2.8 (0.8)	2.8 (0.7)	*F* < 1
Trail-Making Test A	35.4 sec (10.7)	39.2 sec (12.3)	36.4 sec (8.2)	*F*(2, 102) = 1.2, *P* = .30
Trail-Making Test B	93.9 sec (24.5)	96.3 sec (40.5)	97.4 sec (36.3)	*F* < 1

**Table 2:** Performance data of the participant groups separately for the test sessions before (T1) and after (T2) the four-month training interval. Standard deviations are given in parentheses behind the mean values. Significant performance changes within the groups from the first to the second test session are indicated by asterisks (*P* < .05).

Group		Cognitive training	No-contact control	Social control
Response times (ms)	T1	1343 (174)	1364 (184)	1312 (255)
	T2	1428 (202)	1347 (211)	1340 (245)

Missed targets (%)	T1	26.6 (12.1)*	23.7 (12.5)	28.4 (14.1)
	T2	19.9 (12.4)*	24.2 (13.3)	26.1 (14.5)

False alarms (%)	T1	2.8 (9.6)	2.4 (2.7)	2.8 (3.0)
	T2	1.9 (2.5)	6.0 (12.6)	3.2 (4.6)
